# Evaluation of the Unintended Effects of *fad2-1*-Gene-Edited Soybean Line AE15 Seeds

**DOI:** 10.3390/biom16010008

**Published:** 2025-12-19

**Authors:** Ruizhe Wang, Chang Guo, Jihong Zhang, Zhanchao Wang, Wujun Jin, Weixiao Liu

**Affiliations:** 1 Biotechnology Research Institute, Chinese Academy of Agricultural Sciences, Beijing 100081, China; wrz15830282820@gmail.com (R.W.); zhangjihong@caas.cn (J.Z.); wanglujerry155@gmail.com (Z.W.); 2 National Nanfan Research Institute, Chinese Academy of Agricultural Sciences, Sanya 572024, China; 3 College of Bioscience and Biotechnology, Yangzhou University, Yangzhou 225012, China; gc19836611428@gmail.com

**Keywords:** *fad2-1*, gene-edited soybean line AE15 seeds, unintended effects, (DIA)-based proteomic, DEP, co-DEP

## Abstract

A data-independent acquisition (DIA)-based proteomic analysis was performed to evaluate the unintended effects of *fad2-1*-gene-edited soybean line AE15 seeds. A total of 561, 269, and 227 differentially expressed proteins (DEPs) were identified in seeds from three consecutive generations of AE15 soybean, respectively, and were primarily enriched in Kyoto Encyclopedia of Genes and Genomes (KEGG) pathways related to carbon metabolism, protein processing in the endoplasmic reticulum, and proteasome function. Furthermore, eight commonly differentially expressed proteins (co-DEPs) were detected across all three generations of AE15 soybean seeds, among which two—beta-amylase and endoplasmic reticulum (ER) lumen protein-retaining receptor—exhibited consistently upregulated expression. In the wild-type soybean control groups, 1063, 989, and 671 DEPs were identified across the three comparisons (ZhH302E3/ZhH10, ZhH10/ZhH42, and ZhH42/ZhH302E3), among which 71 co-DEPs were observed. These findings indicate that the protein expression profile alterations resulting from *fad2-1* gene editing are considerably less pronounced compared to those caused by natural genetic variation among soybean seeds.

## 1. Introduction

Fatty acid desaturases FAD2-1 and FAD2-2 in soybean are key enzymes responsible for converting oleic to linoleic acid [1,2]. Applying gene editing technologies, such as CRISPR-Cas9, allows for precise modifications to the FAD2 gene, including deletions, insertions, or nucleotide substitutions within the gene sequence. These modifications result in the functional loss of FAD2 alleles, thereby reducing linoleic acid biosynthesis and increasing the oleic acid content in soybean seeds [3,4,5,6]. The high-oleic-acid soybean line AE15 which is edited at the FAD2-1 locus has been granted China’s first safety certificate for agricultural gene-edited biological production. The oleic acid content in the AE15 soybean variety has increased from less than 20% to over 80%, exceeding the levels found in olive oil. Oleic acid demonstrates strong oxidative and thermal stability; therefore, increasing its content in soybean oil can enhance storage stability and provide health benefits. Developing the AE15 soybean line offers new opportunities to advance the soybean industry; high-oleic-acid soybean oil provides superior oil quality and significant market potential, addressing consumer demand for healthy and high-quality edible oils. Furthermore, it presents new cultivar options for farmers, which are expected to improve the profitability of soybean cultivation.

However, while gene editing enhances desirable quality traits in soybeans, it involves modifications to the soybean genome. This raises concerns regarding the safety of gene-edited soybeans for human consumption and use as animal feed. For instance, could gene editing lead to the formation of novel substances, such as new proteins or metabolites? If so, do these substances pose potential safety risks, including toxicity or allergenicity? Although regulatory policies for gene-edited crops vary across countries [7,8], ensuring their safety remains a fundamental requirement. Advancing omics technologies enables comprehensive and quantitative comparative analyses of both the intended and unintended effects associated with gene editing. Currently, data-independent acquisition (DIA)-based proteomic analysis is rapidly evolving into a novel mass spectrometry acquisition methodology [9,10,11], utilizing panoramic scanning, during which a defined range and number of wide mass-to-charge ratio windows are continuously set across each acquisition cycle. All precursor ions within these unbiased fragmentation isolation windows are fragmented and analyzed to obtain secondary mass spectra. Consequently, this approach enables comprehensive peptide detection and deep proteome coverage, offering distinct advantages in terms of data reproducibility, identification sensitivity, and quantitative accuracy, significantly improving data utilization efficiency. Therefore, DIA-based proteomics is particularly suitable for large-scale sample detection and comparative analysis.

In this study, seeds were collected from three consecutive generations of the *fad2-1*-gene-edited soybean line AE15 and its wild-type counterpart ZhH302, as well as two additional wild-type soybean varieties, ZhH10 and ZhH42, which are commonly used as parental lines in novel biotechnology-based breeding programs in China. Using DIA-based proteomic technology, we comprehensively evaluated both intended and unintended effects associated with *fad2-1* gene editing in ZhH302 soybean. As a result, the *fad2-1*-gene-edited soybean AE15 safety profile was systematically assessed.

## 2. Materials and Methods

### 2.1. Plant Materials

We studied the seeds of three consecutive generations of *fad2-1*-gene-edited soybean AE15, the control wild-type soybean ZhH302, as well as wild-type soybean lines ZhH10 and ZhH42. Detailed information regarding the studied soybean lines is listed in Table 1. These studied seeds were harvested at maturity, air-dried, and stored under standard conditions.

### 2.2. PCR-Based Detection of Gene-Edited Soybean Line AE15

Genomic DNA was extracted from soybean seeds using the TIANcombi DNA Lyse & Det PCR Kit (KG203-03, Tiangen, Beijing, China), and a polymerase chain reaction (PCR) was conducted to verify the gene-edited soybean lines’ genetic identities according to their genotypes. Amplification of the *fad2-1* gene fragment was performed using 2× Phanta Max Master Mix (Dye Plus) (P525-03, Vazyme, Nanjing, China), with the primer sequences and locations listed in Supplementary Table S1. The thermal cycling conditions were as follows: initial pre-denaturation at 95 °C for 6 min followed by 35 cycles of denaturation at 95 °C for 30 s, annealing at 60 °C for 30 s, and extension at 72 °C for 1 min, with a final extension at 72 °C for 7 min. The resulting PCR products were sequenced by Sangon Biotech (Shanghai, China) Co., Ltd., with sequence alignment carried out using DNAMAN 9.0 software.

### 2.3. Protein Preparation and Trypsin Digestion

Three technical replicates of soybean line seeds were used for protein profiling analysis in this study. They were ground in liquid nitrogen and incubated in lysis buffer (8 M urea, 50 mM Tris-HCl (pH = 7.4), 1% Triton X-100 (Merck, Shanghai, China), 0.5%protease cocktail (Merck, Shanghai, China) (*v*/*v*), 1% phosphatase inhibitors (Merck, Shanghai, China) (*v*/*v*)), then reduced with 10 mM DL-Dithiothreitol (DTT) (Solarbio, Beijing, China). The suspension was sonicated on ice and centrifuged, then alkylated with 50 mM iodoacetamide (IAM). The total protein concentration was measured using the Bradford method [12]. Trypsin (V5117, Promega (Beijing, China) Biotech Co., Ltd.) and protein from each sample were mixed at ratio of 1:50 (*w*/*w*) and digestion was performed at 37 °C for 16 h, after which the resulting peptide solution was dried via vacuum concentration and resuspended in 200 μL of 0.1% formic acid (FA) in water. Desalting was performed using a 96-well plate (Model Plate NBE ATLAS 96-well, Tecan Group Ltd., Männedorf, Switzerland, 2.5 mg per well) according to the manufacturer’s instructions [13]. The peptide samples were stored at −80 °C until undergoing further analysis via mass spectrometry. For detailed experimental procedures, please refer to the Supplementary Materials.

### 2.4. Mass Spectrometry (MS) Analysis

Mass spectrometric analysis was conducted using an Orbitrap Exploris 480 mass spectrometer (Thermo Scientific, Waltham, MA, USA) in DIA mode [11,14]. Peptide samples were loaded onto a 75 μm ID × 2 cm pre-column (Acclaim PepMap 100, Thermo Scientific, Waltham, MA, USA) connected to a 300 μm ID × 5 mm pre-column (Acclaim PepMap NEO C18, Thermo Scientific, Waltham, MA, USA), followed by undergoing separation on a self-packed reversed-phase C18 analytical column (Reprosil-Pur C18 AQ, 5 μm, Dr. Maisch GmbH, Ammerbuch-Entringen Germany). Peptides retained on the analytical column were eluted using a 90 min linear gradient. Mobile phase A consisted of 0.1% formic acid (FA) in water, while mobile phase B contained 80% acetonitrile (ACN) in water with 0.1% FA. The MS scan range was set to 400–1100 *m*/*z*, with a resolution of 120,000 at *m*/*z* 200, an AGC target of 3.00 × 10^6^, and a maximum injection time of 22 ms. For MS/MS acquisition, 40 variable isolation windows were applied, with collision energies set at 25%, 27%, and 30%; the AGC target was set to 1.00 × 10^6^; the maximum injection time was set to 40 ms; and the electrospray voltage was set to 2.1 kV, with no purge gas used. The ion transfer tube temperature was maintained at 320 °C.

### 2.5. Data Analysis

A project-specific DIA spectral library was constructed using DIA-MS2PEP. The database was based on high-quality and non-redundant Swiss-Prot sequences (UPo00008827_3847_Glycine_max.fasta, 55,857 entries) downloaded in October 2024 with manual curation. A contaminant database was added to account for potential interference from common contaminant proteins, and a decoy database was included for false discovery rate (FDR) estimation. The main database search parameters are shown in the Supplementary Methods. Following sequence database searching, Python (v3.10.4) was utilized for preprocessing quantitative data, including normalization and missing value imputation. Protein abundance values were normalized via median-centering, and missing values were imputed using the row-wise half-minimum method. All data evaluations and statistical analyses were conducted using R software (v4.2.2), with PCA performed using the ‘prcomp’ function and hierarchical clustering implemented via the hierarchical cluster algorithm [15]. To conduct differential expression analysis of the entire proteome, statistical significance was determined using Student’s *t*-test to calculate *p*-values, and fold change was used to assess differential expression magnitude. In this study, proteins were considered significantly upregulated if the *p*-value was <0.05 and the fold change was ≥2.0, and significantly downregulated if the *p*-value was <0.05 and the fold change was ≤0.50 [16,17,18]. KEGG pathway annotation and enrichment analysis were conducted on the differentially expressed proteins using the ‘clusterProfiler’ package (v4.6.0) and related R packages [19].

### 2.6. qRT-PCR

Approximately 1.0 g of seeds per soybean line was ground in liquid nitrogen and used for total RNA extraction with Trizol Reagent (15596018, Invitrogen, Carlsbad, CA, USA), following the manufacturer’s instructions. RNA integrity was assessed by agarose gel electrophoresis. Subsequently, 1.0 μg of RNA was reverse-transcribed using ReverTra Ace qPCR RT Kit (FSQ-101, Toyobo, Shanghai, China) according to the manufacturer’s protocol. Gene-specific primers for quantitative real-time PCR (qRT-PCR) are listed in Supplementary Table S2 and were designed using Primer Premier 5.0. qRT-PCR was carried out with three technical replicates using the SYBR Green Realtime PCR Master Mix (QPK-212, Toyobo) in accordance with the manufacturer’s instructions. All reactions were conducted on a CFX96 Real-Time PCR System (Bio-Rad, Hercules, CA, USA). The qRT-PCR data were analyzed using the 2^−ΔΔCT^ relative quantification method [20]. *Actin* expression was quantified as an internal control.

### 2.7. New ORF Prediction, Sequence Alignment, and Protein Structure Prediction

Potential novel open reading frames (ORFs) generated by *fad2-1* gene editing were predicted using online tools available through the NCBI platform. Sequence alignment of the newly generated ORFs resulting from *fad2-1* gene editing was conducted using DNAMAN software. Protein structure modeling of both the wild-type FAD2-1 and the newly predicted ORFs derived from *fad2-1* gene editing was performed using the Alpha Fold 3.0 online server [21,22,23], with the resulting structures visualized using PyMOL3.1.3.1 software [24,25].

## 3. Results

### 3.1. Soybean Line Confirmation

**Figure 1:**
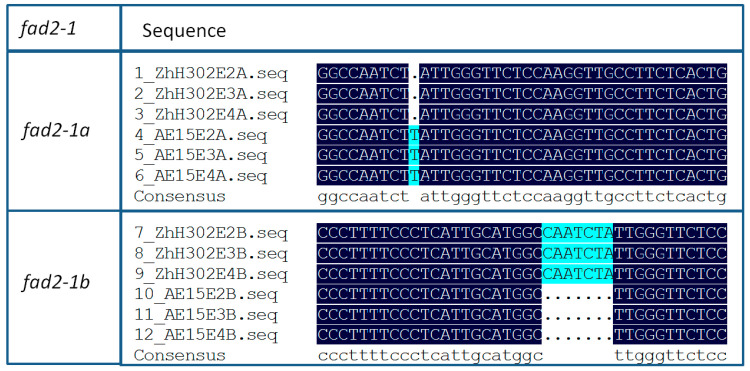
*fad2-1* gene sequence alignment in three consecutive *fad2-1*-gene-edited soybean line AE15 generations compared to ZhH302. There was a thymine (T) insertion immediately after nucleotide position 277 in the edited *fad2-1a* on the AE15 line’s A genome across all three generations; a seven-base deletion (CAATCTA) was detected following nucleotide position 271 in the edited *fad2-1b* on the AE15 line’s B genome across all generations. Complete sequence alignment data are provided in Figure S2.

In this study, we analyzed seeds from three consecutive *fad2-1*-gene-edited soybean line AE15 generations, the control wild-type soybean ZhH302, and two additional wild-type lines, ZhH10 and ZhH42 (Table 1). PCR amplification of *fad2-1* fragments (1–620 bp of *fad2-1a* and 23–647 bp of *fad2-1b*) (Figure S1), sequencing, and sequence alignment analyses revealed a thymine (T) insertion immediately after nucleotide position 277 in the AE15 line’s edited A genome across all three generations, whereas a seven-base deletion (CAATCTA) was detected following nucleotide position 271 in the same line’s edited B genome (Figure 1 and Figure S2).

### 3.2. Protein Profiling of Soybean Seeds

**Figure 2:**
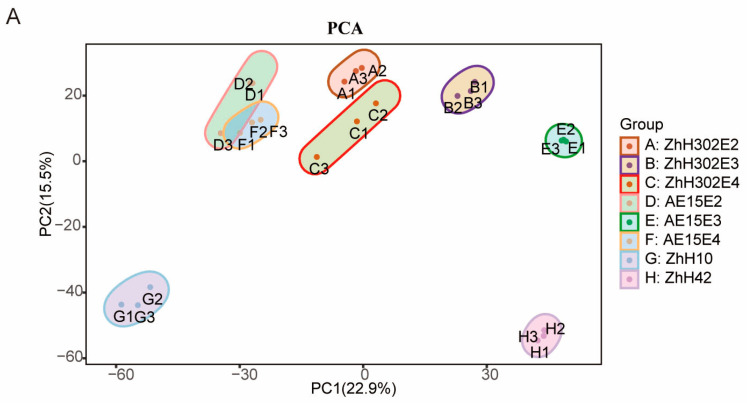

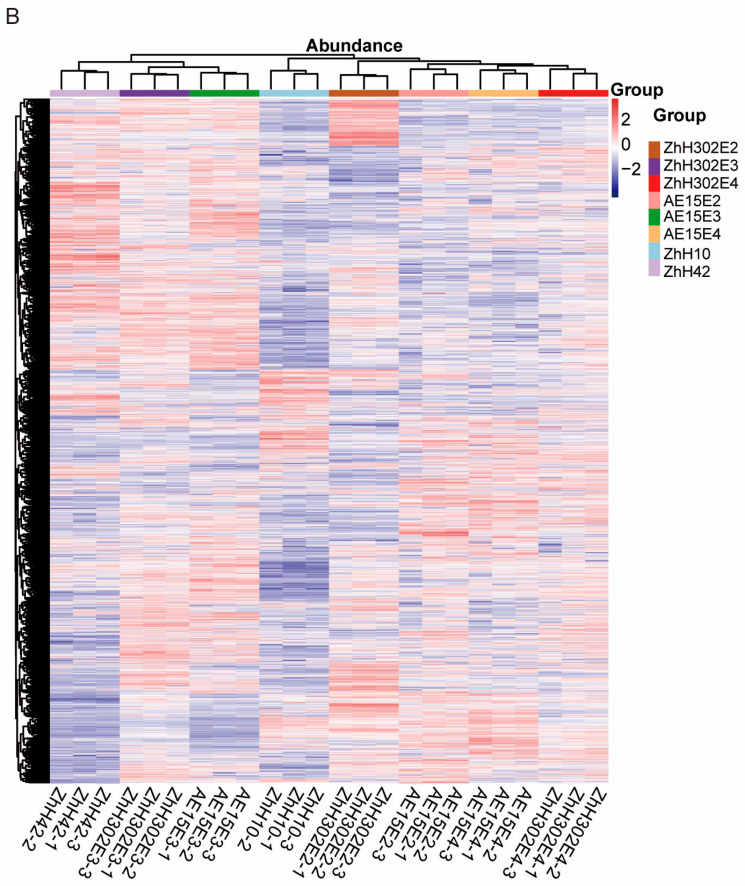
Proteomic profiling of seeds from the studied soybean lines. (**A**) Protein expression profile principal component analysis (PCA) in eight soybean lines’ seeds. The score plot displays the first two principal components and their corresponding explained variances. (**B**) Cluster map comparing protein expression patterns, where red indicates relatively high expression, blue indicates relatively low expression, and white indicates similar expression levels between lines. All mass spectrometry (MS) data were normalized prior to cluster analysis.

A total of 5493 proteins were identified across three technical replicates after filtering for at least one unique peptide, of which between 4512 and 5254 were quantified across the eight soybean lines examined (Supplementary Table S3). Principal component analysis (PCA) identified PC1 and PC2 as the two primary components, accounting for 22.90% and 15.50% of the variance, respectively (Figure 2A). Protein abundance profile hierarchical clustering indicated that the eight soybean lines formed two distinct clusters: one included AE15E4 and ZhH302E4, which exhibited the highest similarity in protein expression patterns, followed by AE15E2, ZhH302E2, and ZhH10; the second comprised AE15E3 and ZhH302E3, which showed greater similarity to each other than to ZhH42 (Figure 2B).

### 3.3. DEP Detection in Soybean Seeds

The number and regulatory direction of DEPs across comparison groups are summarized in Table 2. In the AE15E2 versus ZhH302E2 comparison, 561 DEPs were identified, including 292 upregulated and 269 downregulated proteins. Comparing AE15E3 and ZhH302E3 revealed 269 DEPs, with 78 upregulated and 191 downregulated, and AE15E4 and ZhH302E4 revealed 227 DEPs, with 101 upregulated and 126 downregulated. All DEPs from the three AE15/ZhH302 comparisons are listed in Supplementary Tables S4–S6. Additionally, 1063 DEPs were identified in ZhH302E3 versus ZhH10, 989 in ZhH10 versus ZhH42, and 671 in ZhH302E3 versus ZhH42 (DEPs provided in Supplementary Tables S7–S9). Comparisons among ZhH302’s developmental generations (ZhH302E2 vs. ZhH302E3, ZhH302E3 vs. ZhH302E4, and ZhH302E4 vs. ZhH302E2) yielded 442, 242, and 545 DEPs, respectively. Similarly, comparisons within AE15’s developmental generations (AE15E2 vs. AE15E3, AE15E3 vs. AE15E4, and AE15E2 vs. AE15E4) identified 623, 666, and 214 DEPs, respectively (DEPs detailed in Supplementary Tables S10–S15).

### 3.4. KEGG Pathway Enrichment Analysis of the Identified DEPs in AE15 Soybean Seeds

**Figure 3:**
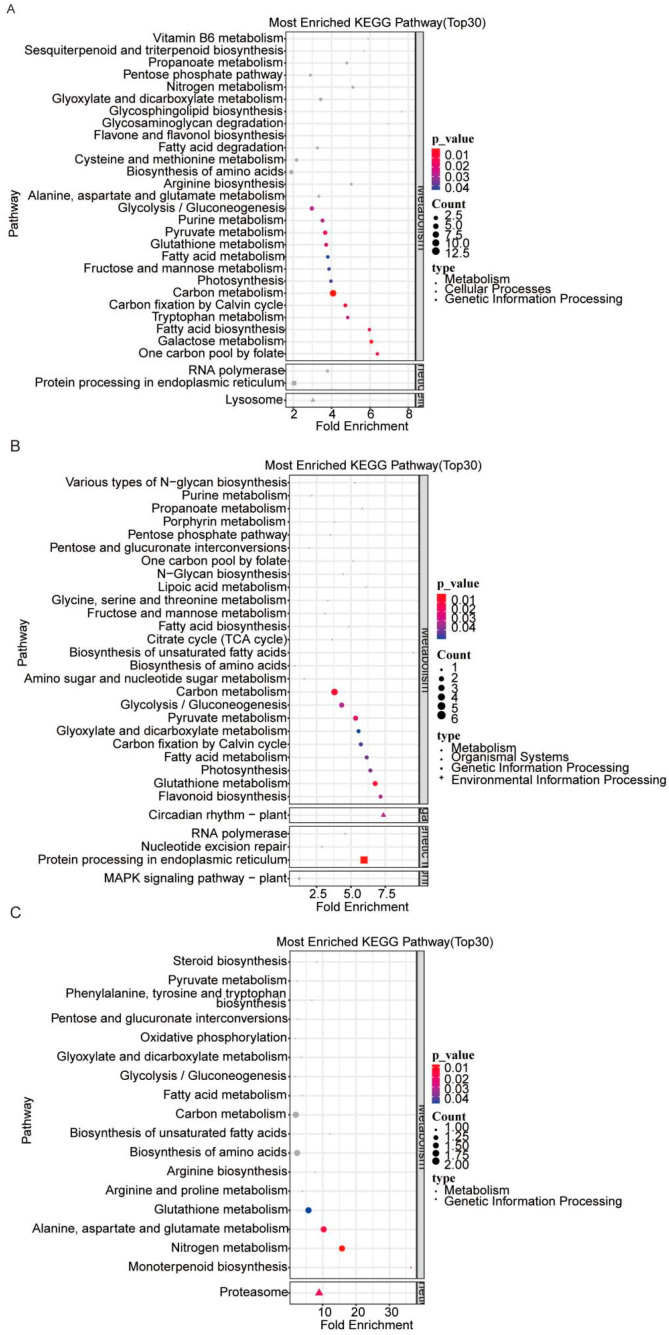
KEGG pathway enrichment analysis of DEPs in AE15E2 versus ZhH302E2 (**A**), AE15E3 versus ZhH302E3 (**B**), and AE15E4 versus ZhH302E4 (**C**). The top 30 enriched pathways were selected based on ascending *p*-values, with the lowest 30 values chosen for visualization in bubble plots. In the color scale, redder shades indicate smaller (more significant) *p*-values, while bluer shades reflect larger (less significant) *p*-values.

The KEGG pathway enrichment analysis results indicated that DEPs from the three AE15/ZhH302 comparisons were predominantly associated with carbon metabolism. Specifically, in the AE15E2/ZhH302E2 comparison, DEPs were enriched in pathways related to protein processing in the endoplasmic reticulum and lysosome (Figure 3A). In the AE15E3/ZhH302E3 comparison, significant enrichment was observed in the circadian rhythm—plant, protein processing in the endoplasmic reticulum, and MAPK signaling pathway—plant (Figure 3B). In contrast, DEPs in the AE15E4/ZhH302E4 comparison were primarily enriched in proteasome-related pathways (Figure 3C).

### 3.5. Identifying Co-DEPs and FAD2-1 in Soybean Seeds

**Figure 4:**
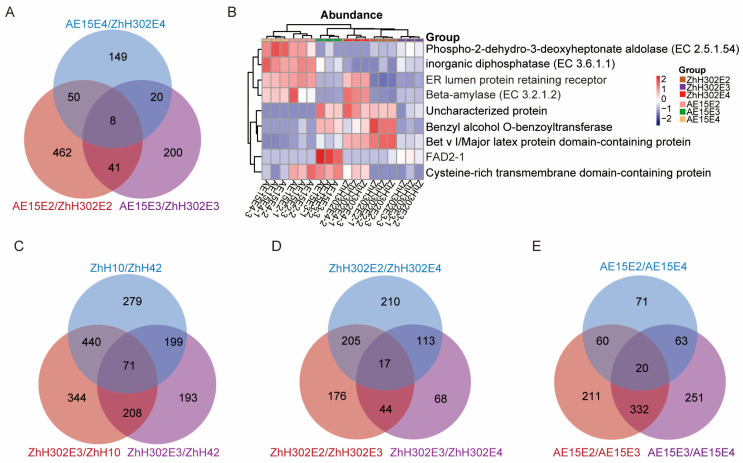
co-DEPs identified in various comparison groups. (**A**) Venn diagram illustrating number of overlapping DEPs from pairwise comparisons among three AE15/ZhH302 groups. (**B**) Expression trends of eight co-DEPs and FAD2-1, where red indicates relatively high expression, blue indicates relatively low expression, and white indicates similar expression levels between lines. Venn diagrams show number of overlapping DEPs from pairwise comparisons among three wild-type soybean lines ZhH302E3, ZhH10, and ZhH42 (**C**); among three generations of ZhH302 (**D**); and among three generations of AE15 (**E**).

A total of eight DEPs were consistently identified across the three AE15/ZhH302 comparison groups (Figure 4A). FAD2-1 was significantly downregulated in AE15E3 and AE15E4 soybean seeds and downregulated in AE15E2-R3 relative to ZhH302E2-R3 (Supplementary Table S16). These commonly differentially expressed proteins (co-DEPs), together with FAD2-1, were significantly enriched in pathways related to fatty acid metabolism and unsaturated fatty acid biosynthesis (Figure S3A). Among the identified co-DEPs, beta-amylase and ER lumen protein-retaining receptor exhibited upregulated expression across all three generations of AE15 soybean seeds. In contrast, the expression patterns of six other proteins—cysteine-rich transmembrane domain-containing protein, inorganic diphosphatase (EC 3.6.1.1), phospho-2-dehydro-3-deoxyheptonate aldolase (EC 2.5.1.54), benzyl alcohol O-benzoyltransferase, Bet v I/major latex protein domain-containing protein, and uncharacterized protein (A0A0R0IT88)—were inconsistent across the three generations (Figure 4B, Table 3).

Seventy-one co-DEPs were identified through pairwise comparisons among the three wild-type soybean lines ZhH302E3, ZhH10, and ZhH42 (Figure 4C), and were significantly enriched in the porphyrin metabolism and flavonoid biosynthesis pathways (Figure S3B). Additionally, 17 co-DEPs were consistently identified across the three groups involving pairwise comparisons of the three ZhH302 generations (Figure 4D), and were significantly enriched in glutathione metabolism (Figure S3C). Similarly, 20 co-DEPs were consistently identified across the three groups involving pairwise comparisons of the three AE15 generations (Figure 4E), and were significantly enriched in protein processing in the endoplasmic reticulum (Figure S3D).

### 3.6. Selected Co-DEPs Further Analyzed by qRT-PCR

**Figure 5:**
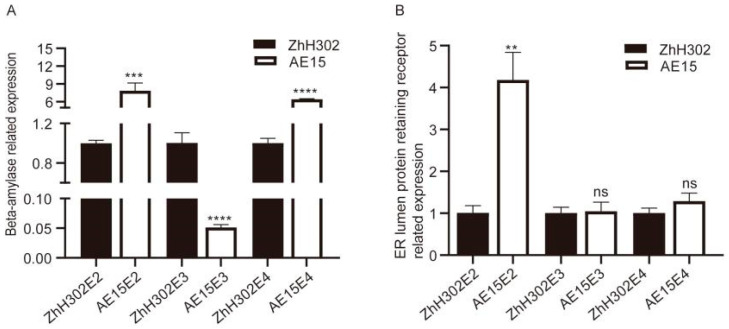
qRT-PCR analysis of selected co-DEPs’ gene expression patterns in the three AE15/ZhH302 comparison groups. (**A**) Beta-amylase; (**B**) ER lumen protein-retaining receptor. Error bars represent standard deviation (SD) across three biological replicates. Asterisks denote statistically significant differences compared to ZhH302, as determined by Student’s *t*-test (** *p* < 0.01, *** *p* < 0.001, **** *p* < 0.0001, ns, no significant difference).

Two upregulated co-DEPs—beta-amylase and ER lumen protein-retaining receptor—were selected for quantitative reverse transcription PCR (qRT-PCR) analysis to validate their expression patterns at the transcriptional level. The transcriptional profiles of these genes are presented in Figure 5. Compared with the wild-type ZhH302, beta-amylase was significantly upregulated in AE15E2 and AE15E4 but significantly downregulated in AE15E3 (Figure 5A). The ER lumen protein-retaining receptor was significantly upregulated in AE15E2, with no significant changes observed in AE15E3 and AE15E4 (Figure 5B).

### 3.7. FAD2-1 in Studied Soybean Seeds

**Figure 6:**
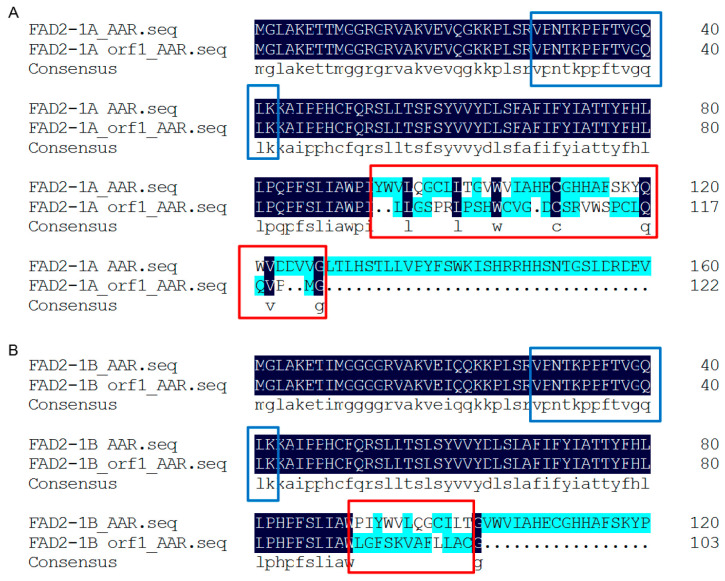

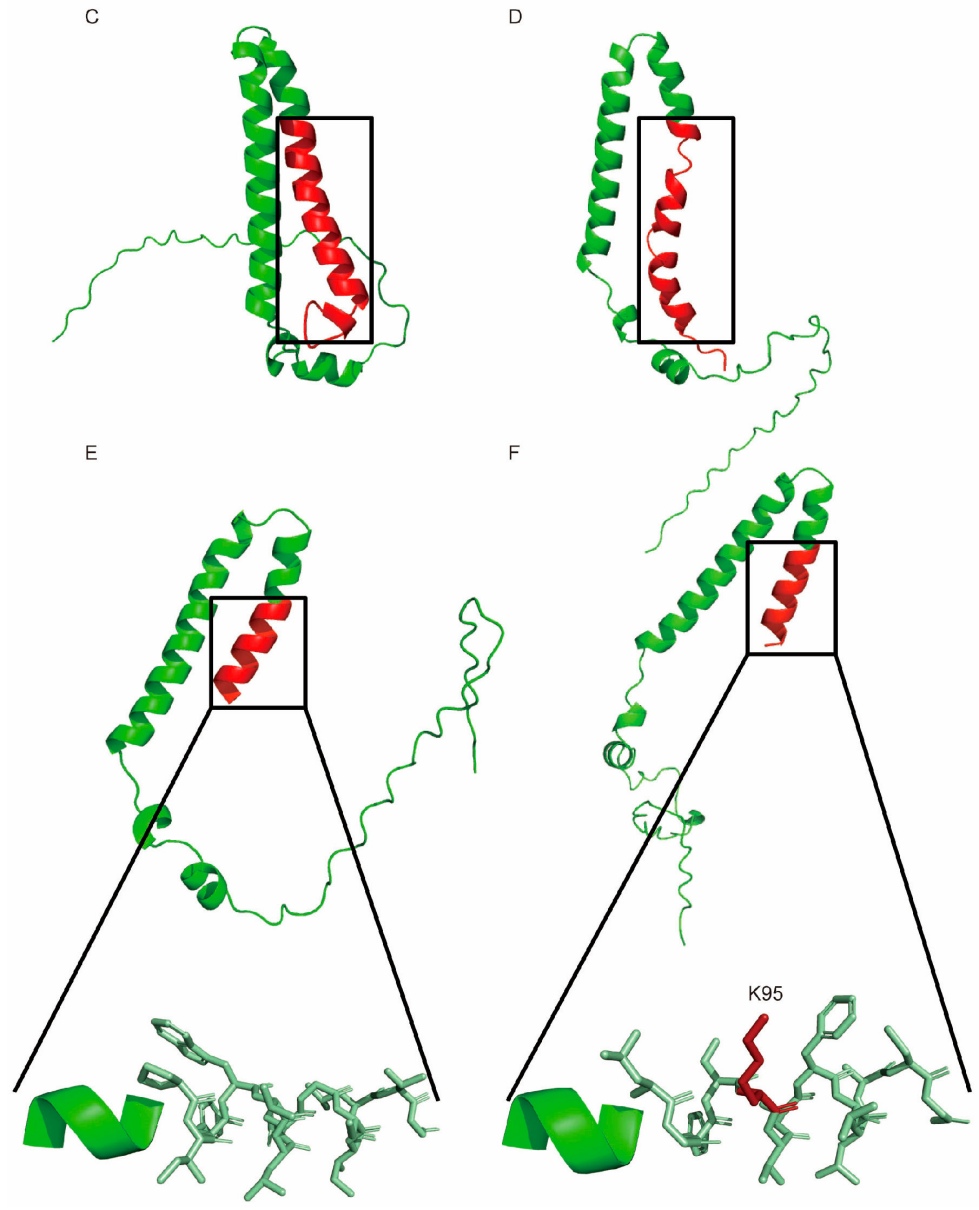
Characterization of FAD2-1 in soybean. FAD2-1A (**A**) and FAD2-1B (**B**) amino acid sequence alignments before and after gene editing are shown. The blue box highlights the unique FAD2-1 peptide identified in this study, while the red box indicates amino acid sequence variations in FAD2-1 between the wild-type (ZhH302) and edited (AE15) lines. The predicted spatial structures are displayed for FAD2-1A in wild-type ZhH302 (**C**), FAD2-1A edited in AE15 (**D**), wild-type FAD2-1B in ZhH302 (**E**), and FAD2-1B edited in AE15 (**F**). Conserved regions before and after editing are shown in green, whereas altered regions are indicated in red.

As represented by the unique peptide “VPNTKPPFTVGQLK” located upstream of the editing site (Figure 6A,B), FAD2-1 was found to be significantly more abundant in ZhH302E2-R3, ZhH302E3, and ZhH302E4 compared to AE15E2, AE15E3, and AE15E4 (Table S16), as revealed by MS analysis. The C-terminal sequence alteration in the FAD2-1 truncation generated by gene editing resulted in a more flexible conformation of the FAD2-1A variant (Figure 6C,D), while FAD2-1B introduced a novel cleavage site at lysine residue 95 (K95) (Figure 6E,F).

## 4. Discussion

In this study, a data-independent acquisition (DIA)-based proteomic analysis was conducted to evaluate the *fad2-1*-gene-edited AE15 soybean seed safety profile. Compared with label-based quantitative proteomic methods, DIA-based proteomics eliminates potential interference caused by labeling reagents and offers greater cost efficiency. A total of 5493 proteins were identified through DIA-based proteomic profiling, with approximately 4% to 10% classified as DEPs across three consecutive AE15 soybean seed generations, which is significantly lower than the proportion observed in three wild-type soybean seeds (approximately 12% to 19%). Importantly, none of these DEPs were found to correspond to novel toxins or allergens, a finding consistent with results from previous studies on genetically modified (GM) crops [17,26,27,28,29] and gene edit crops [30].

The identified DEPs across the three AE15 soybean seed generations were significantly enriched in pathways associated with protein processing in the endoplasmic reticulum and proteasome activity. Moreover, a decreasing trend in the DEP number was observed across successive AE15 soybean seed generations; however, further investigation involving additional generations or diverse experimental materials is required to determine whether this trend reflects a genuine biological pattern or occurs by chance.

The results indicated that the co-DEPs identified across three consecutive AE15 soybean seed generations were also significantly reduced compared to those identified in three wild-type soybean seeds, as well as across three consecutive ZhH302 and AE15 soybean seed generations. These co-DEPs from different genetic backgrounds were associated with distinct KEGG pathways and performed diverse biological functions. Among the co-DEPs identified across three consecutive AE15 soybean seed generations, both beta-amylase and ER lumen protein-retaining receptor were consistently upregulated in all generations when compared to wild-type ZhH302, an observation inconsistent with their corresponding transcriptional expression levels. It should be noted that gene expression encompasses multiple stages, including transcription, post-transcriptional modification, translation, and post-translational modification, all of which contribute to mature proteins forming. Protein expression and maturation represent complex biological processes that are also subject to intrinsic regulatory mechanisms [31,32,33].

MS analysis detected FAD2-1 to be significantly more abundant in ZhH302E2-R3, ZhH302E3, and ZhH302E4 compared to AE15E2, AE15E3, and AE15E4. This observation may be attributed to structural modifications in the truncated FAD2-1 product potentially generated following gene editing. Specifically, FAD2-1A’s structure appears to adopt a more loosely folded conformation, whereas FAD2-1B’s variant sequence does not exhibit significant structural alterations but introduces potential protease cleavage sites.

## 5. Conclusions

Soybean seed proteomic profiling indicated that the alterations in protein expression profiles resulting from *fad2-1* gene editing were considerably less pronounced than those observed in soybeans with distinct genetic backgrounds. DEPs identified across the three AE15 soybean seed generations were not classified as novel unintended proteins, toxins, or allergens; only variations in expression levels were detected. These DEPs were primarily associated with carbon metabolism, protein processing in the endoplasmic reticulum, and proteasome function pathways. Notably, the transcriptional and translational levels of the two upregulated co-DEPs exhibited inconsistency and did not display consistent patterns across the three AE15 soybean seed generations.

## Figures and Tables

**Table 1:** Summary of the soybean lines analyzed in this study.

Sample	Genotype/Authorized Number
ZhH302E2	wild-type(Yudou22xAg31)/Wanshendou2018006
ZhH302E3	
ZhH302E4	
AE15E2	*fad2*-*1* edited in ZhH302
AE15E3	
AE15E4	
ZhH10	wild-type(Wenfeng7xLudou4)/(96)jingshenjingzi2
ZhH42	wild-type(Youchu4xJindou33)/Guoshengdou2007002

**Table 2:** Summary of the number and regulatory direction of DEPs in various comparison groups.

Comparison Group	No. of DEPs	No. Upregulated	No. Downregulated
AE15E2/ZhH302E2	561	292	269
AE15E3/ZhH302E3	269	78	191
AE15E4/ZhH302E4	227	101	126
ZhH302E3/ZhH10	1063	901	162
ZhH10/ZhH42	989	407	582
ZhH302E3/ZhH42	671	108	563
ZhH302E2/ZhH302E3	442	154	288
ZhH302E3/ZhH302E4	242	151	91
ZhH302E4/ZhH302E2	545	305	240
AE15E2/AE15E3	623	349	274
AE15E3/AE15E4	666	292	374
AE15E4/AE15E2	214	95	119

**Table 3:** Eight co-DEPs’ expression regulation patterns across the three AE15/ZhH302 comparison groups.

ID	Name	AE15/ZhH302 Comparison Groups		
		E2	E3	E4
GLYMA_09G168300_A0A0R4J443	Beta-amylase (EC 3.2.1.2)	Up	Up	Up
LOC100790733 GLYMA_03G084600_I1JM56	ER lumen protein-retaining receptor	Up	Up	Up
GLYMA_15G256000_K7MDY4	Cysteine-rich transmembrane domain-containing protein	Up	Up	Down
GLYMA_13G132400_A0A0R0GZH8	Inorganic diphosphatase (EC 3.6.1.1)	Up	Down	Up
GLYMA_06G101700_I1K9W3	Phospho-2-dehydro-3-deoxyheptonate aldolase (EC 2.5.1.54)	Up	Down	Down
GLYMA_03G244600_I1JRK7	Benzyl alcohol O-benzoyltransferase	Down	Up	Down
LOC100527208 GLYMA_15G218900_A0A0R0GE22	Bet v I/Major latex protein domain-containing protein	Down	Up	Down
GLYMA_08G274500_A0A0R0IT88	Uncharacterized protein	Down	Up	Down

## Data Availability

All authors confirm that all data and materials support their published claims and comply with field standards. These data and materials are provided within this article and its Supplementary Materials.

## Supplementary Materials

The following supporting information can be downloaded at https://www.mdpi.com/article/10.3390/biom16010008/s1: Figure S1: PCR amplification of *fad2-1* fragments (1–620 bp of *fad2-1a* and 23–647 bp of *fad2-1b*). M, DNA marker; 1, ZhH302E2; 2, ZhH302E3; 3, ZhH302E4; 4, AE12E2; 5, AE12E3; 6, AE12E4; 7, ZhH302E2; 8, ZhH302E3; 9, ZhH302E4; 10, AE12E2; 11, AE12E3; 12, AE12E4. Figure S2: *fad2-1* fragments sequence alignment in three consecutive generations of the *fad2-1*-gene-edited AE15 soybean line compared to ZhH302. A, *fad2-1a*; B, *fad2-1b.* Figure S3: KEGG pathway enrichment analysis of co-DEPs identified from pairwise comparisons among the three AE15/ZhH302 groups (A); wild-type soybean lines ZhH302E3, ZhH10, and ZhH42 (B); three consecutive generations of ZhH302 (C); and AE15 (D). Table S1: Primers for confirm the genetic identity of the gene-edited soybean AE15. Table S2: The sequences of gene-specific primers for qRT-PCR. Table S3: Summary of the proteins identified in this study. Tables S4–S6: DEPs identified from AE15E2/ZhH302E2, AE15E3/ZhH302E3, and AE15E4/ZhH302E4. Tables S7–S9: DEPs identified from ZhH302E3/ZhH10, ZhH10/ZhH42, and ZhH42/ZhH302E3. Tables S10–S12: DEPs identified from ZhH302E2/ZhH302E3, ZhH302E3/ZhH302E4, and ZhH302E4/ZhH302E2. Tables S13–S15: DEPs identified from AE15E2/AE15E3, AE15E3/AE15E4, and AE15E4/AE15E2. Table S16: FAD2-1 detected by MS analysis.

## Abbreviations

The following abbreviations are used in this manuscript:

FAD2-1	Fatty acid desaturase 2-1
DIA	Data-independent acquisition
FC	Fold change
DEPs	Differentially expressed proteins
co-DEPs	Commonly differentially expressed proteins
KEGG	Kyoto Encyclopedia of Genes and Genomes
qRT-PCR	Quantitative real-time PCR
MS	Mass spectrometry
ZhH	Zhonghuang
AE15	*fad2-1*-gene-edited soybean line AE15

## References

[1] Dar A.A., Choudhury A.R., Kancharla P.K., Arumugam N. (2017). The FAD2 Gene in Plants: Occurrence, Regulation, and Role. Front. Plant Sci..

[2] Lakhssassi N., Zhou Z., Liu S., Colantonio V., AbuGhazaleh A., Meksem K. (2017). Characterization of the FAD2 Gene Family in Soybean Reveals the Limitations of Gel-Based TILLING in Genes with High Copy Number. Front. Plant Sci..

[3] Pham A.T., Lee J.D., Shannon J.G., Bilyeu K.D. (2011). A novel FAD2-1 A allele in a soybean plant introduction offers an alternate means to produce soybean seed oil with 85% oleic acid content. Theor. Appl. Genet..

[4] Pham A.T., Lee J.D., Shannon J.G., Bilyeu K.D. (2010). Mutant alleles of FAD2-1A and FAD2-1B combine to produce soybeans with the high oleic acid seed oil trait. BMC Plant Biol..

[5] Haun W., Coffman A., Clasen B.M., Demorest Z.L., Lowy A., Ray E., Retterath A., Stoddard T., Juillerat A., Cedrone F. (2014). Improved soybean oil quality by targeted mutagenesis of the fatty acid desaturase 2 gene family. Plant Biotechnol. J..

[6] Zhang W., Xu W., Xu Y., Zhang H., Liu X., Cui X., Chen X., Chen H. (2023). Creation of high oleic acid soybean lines by CRISPR/Cas9. J. Jiangsu Agric..

[7] Entine J., Felipe M.S.S., Groenewald J.H., Kershen D.L., Lema M., McHughen A., Nepomuceno A.L., Ohsawa R., Ordonio R.L., Parrott W.A. (2021). Regulatory approaches for genome edited agricultural plants in select countries and jurisdictions around the world. Transgenic Res..

[8] Turnbull C., Lillemo M., Hvoslef-Eide T.A.K. (2021). Global Regulation of Genetically Modified Crops Amid the Gene Edited Crop Boom—A Review. Front. Plant Sci..

[9] Hou J., Wang J., Yang F., Xu T. (2022). DIA-MS2pep: A library-free framework for comprehensive peptide identification from data-independent acquisition data. Biophys. Rep..

[10] Demichev V., Messner C.B., Vernardis S.I., Lilley K.S., Ralser M. (2020). DIA-NN: Neural networks and interference correction enable deep proteome coverage in high throughput. Nat. Methods.

[11] Lou R., Shui W. (2024). Acquisition and Analysis of DIA-Based Proteomic Data: A Comprehensive Survey in 2023. Mol. Cell. Proteom..

[12] Bradford M.M. (1976). A rapid and sensitive method for the quantitation of microgram quantities of protein utilizing the principle of protein-dye binding. Anal. Biochem..

[13] Rappsilber J., Mann M., Ishihama Y. (2007). Protocol for micro-purification, enrichment, pre-fractionation and storage of peptides for proteomics using StageTips. Nat. Protoc..

[14] Lou R., Cao Y., Li S., Lang X., Li Y., Zhang Y., Shui W. (2023). Benchmarking commonly used software suites and analysis workflows for DIA proteomics and phosphoproteomics. Nat. Commun..

[15] Kimes P.K., Liu Y., Neil Hayes D., Marron J.S. (2017). Statistical significance for hierarchical clustering. Biometrics.

[16] Zeng W.Y., Sun Z.D., Cai Z.Y., Chen H.Z., Lai Z.G., Yang S.Z., Tang X.M. (2017). Proteomic analysis by iTRAQ-MRM of soybean resistance to Lamprosema Indicate. Bmc Genomics.

[17] Wang L.M., Wang X.C., Jin X., Jia R.Z., Huang Q.X., Tan Y.H., Guo A.P. (2015). Comparative proteomics of Bt-transgenic and non-transgenic cotton leaves. Proteome Sci..

[18] Liu Y.B., Zhang Y.X., Song S.Q., Li J.S., Stewart C.N., Wei W., Zhao Y.J., Wang W.Q. (2015). A proteomic analysis of seeds from Bt-transgenic Brassica napus and hybrids with wild B. juncea. Sci. Rep..

[19] Kanehisa M., Goto S., Sato Y., Furumichi M., Tanabe M. (2012). KEGG for integration and interpretation of large-scale molecular data sets. Nucleic Acids Res..

[20] Livak K.J., Schmittgen T.D. (2001). Analysis of relative gene expression data using real-time quantitative PCR and the 2(T)(-Delta Delta C) method. Methods.

[21] Abramson J., Adler J., Dunger J., Evans R., Green T., Pritzel A., Ronneberger O., Willmore L., Ballard A.J., Bambrick J. (2024). Accurate structure prediction of biomolecular interactions with AlphaFold 3. Nature.

[22] Varadi M., Anyango S., Deshpande M., Nair S., Natassia C., Yordanova G., Yuan D., Stroe O., Wood G., Laydon A. (2022). AlphaFold Protein Structure Database: Massively expanding the structural coverage of protein-sequence space with high-accuracy models. Nucleic Acids Res..

[23] Jumper J., Evans R., Pritzel A., Green T., Figurnov M., Ronneberger O., Tunyasuvunakool K., Bates R., Zidek A., Potapenko A. (2021). Highly accurate protein structure prediction with AlphaFold. Nature.

[24] Chen Y., Zhang H., Wang W., Shen Y., Ping Z. (2024). Rapid generation of high-quality structure figures for publication with PyMOL-PUB. Bioinformatics.

[25] Rigsby R.E., Parker A.B. (2016). Using the PyMOL application to reinforce visual understanding of protein structure. Biochem. Mol. Biol. Educ..

[26] Balsamo G.M., Cangahuala-Inocente G.C., Bertoldo J.B., Terenzi H., Arisi A.C.M. (2011). Proteomic Analysis of Four Brazilian MON810 Maize Varieties and Their Four Non-Genetically-Modified Isogenic Varieties. J. Agr. Food Chem..

[27] Fu W., Wang C.G., Xu W.J., Zhu P.Y., Lu Y., Wei S., Wu X.Y., Wu Y.P., Zhao Y.Q., Zhu S.F. (2019). Unintended effects of transgenic rice revealed by transcriptome and metabolism. Gm. Crops Food-Biotechnol. Agric. Food Chain.

[28] Gong C.Y., Li Q., Yu H.T., Wang Z.Z., Wang T. (2012). Proteomics Insight into the Biological Safety of Transgenic Modification of Rice As Compared with Conventional Genetic Breeding and Spontaneous Genotypic Variation. J. Proteome Res..

[29] Liu W.X., Xu W.T., Li L., Dong M., Wan Y.S., He X.Y., Huang K.L., Jin W.J. (2018). iTRAQ-based quantitative tissue proteomic analysis of differentially expressed proteins (DEPs) in non-transgenic and transgenic soybean seeds. Sci. Rep..

[30] Liu X.-J., Xing B., Wang M.-Y., Li X.-M., Wang X.-J., Wang Z.-X. (2023). Transcriptional and proteomic analysis. GM Crops Food.

[31] Pedrazzini E., Vitale A. (2018). Protein Biosynthesis and Maturation in the ER. Plant Endoplasmic Reticulum.

[32] Vitale A., Boston R.S. (2008). Endoplasmic reticulum quality control and the unfolded protein response: Insights from plants. Traffic.

[33] Liu J.X., Howell S.H. (2016). Managing the protein folding demands in the endoplasmic reticulum of plants. New Phytol..

