# Supplementary material for: Evaluation of the Unintended Effects of fad2-1-Gene-Edited Soybean Line AE15 Seeds
Source: Biomolecules. 2025 Dec 19;16(1):8. doi: 10.3390/biom16010008 (PMC12838885; doi:10.3390/biom16010008)
Supplement: Supplementary file 1 [file biomolecules-16-00008-s001.zip › sup1108 short Revised 1204.pdf]

Evaluating the Unintended Effects of *fad2-1*-Gene-Edited Soybean Line AE15 Seeds

Ruizhe Wang [1,†], Chang Guo [2,†], Jihong Zhang [1], Zhanchao Wang [1], Wujun Jin [1,3,*] and Weixiao Liu [1,*]

[1] Biotechnology Research Institute, Chinese Academy of Agricultural Sciences, Beijing 100081, China; wrz15830282820@gmail.com (R.W.); zhangjihong@caas.cn (J.Z.); wanglujerry155@gmail.com (Z.W.)

[2] College of Bioscience and Biotechnology, Yangzhou University, Yangzhou 225012, China; gc19836611428@gmail.com

[3] National Nanfan Research Institute, Chinese Academy of Agricultural Sciences, Sanya 572024, China

[*] Correspondence: jinwujun@caas.cn (W.J.); liuweixiao@caas.cn (W.L.)

[†] These authors contributed equally to this work.

Materials and Methods

1 PCR-based detection of gene-edited soybean

The sequences of the primers used to detect the gene-edited soybean AE15 are provided in Supplementary Table S1.

Table S1 Primers for confirm the genetic identity of the gene-edited soybean AE15.

**Primer**	**Sequence (5'-3')**	Location
***fad2-1-a* F**	ATGGGTCTAGCAAAGGAAAC	1-20
***fad2-1-a* R**	TATCATAGGGTCTACCAGAG	601-620
***fad2-1-b* F**	TAATGGGAGGTGGAGGCCGT	23-42
***fad2-1-b* R**	AGCATAAGGGTGGTAGTGGC	628-647

2. Protein Preparation and Trypsin Digestion

For each sample, an appropriate amount of soybean seeds was collected and ground into a fine powder under liquid nitrogen. Lysis buffer (8 M urea, 50 mM Tris-HCl, pH 7.4, 1% Triton X-100, 0.5% protease cocktail [v/v], and 1% phosphatase inhibitors [v/v]) was added, and the mixture was vortexed thoroughly to ensure complete solubilization. The homogenate was transferred to a grinding tube for further mechanical disruption. After centrifugation at 15,000 × g at 4 °C for 5 minutes, the supernatant was carefully collected and transferred to a fresh microcentrifuge tube. Protein concentration was determined using the BCA Protein Assay Kit (Thermo Scientific™, Waltham, MA, USA). For subsequent processing, 600 μg of protein per sample was reduced by addition of 10 mM dithiothreitol (DTT), followed by incubation at room temperature for 1 hour. Subsequently, alkylation was performed by adding 50 mM iodoacetamide (IAM), and the samples were incubated in the dark at room temperature for 30 minutes. Excess IAM was quenched by adding an additional aliquot of DTT, followed by a 30-minute incubation at room temperature. Next, 60 μL of SP3 beads were added to each sample, and the mixture was incubated at room temperature for 15 minutes to facilitate efficient protein binding. Trypsin was added at a mass ratio of 1:50 (trypsin:protein) (Promega, Madison, WI, USA), and digestion was carried out at 37 °C for 16 hours. Following enzymatic cleavage, the resulting peptide mixture was dried by vacuum concentration. The dried peptides were resuspended in 200 μL of 0.1% formic acid (FA) in water. Desalting was performed using a 96-well solid-phase extraction plate (NBE ATLAS 96-well, 2.5 mg per well; Thermo Scientific™), according to the manufacturer's instructions. The column was pre-equilibrated sequentially with 600 μL of 0.1% FA in acetonitrile (ACN), 600

μL of 50% ACN in 0.1% FA, and 600 μL of 0.1% FA in water, with flow-through discarded after each step. Samples were loaded onto the corresponding wells and subjected to pressure filtration three times; filtrates were collected and labeled accordingly. The column was washed twice with 600 μL of 0.1% FA in water, discarding the wash fractions. A collection plate was placed beneath the column, and peptides were eluted twice with 100 μL of 50% ACN in 0.1% FA per elution. Eluates were combined, labeled, and concentrated using a vacuum concentrator under the following conditions: V-AQ interface, 45 °C. The final dried peptide samples were stored at –80 °C until analysis by mass spectrometry.

3. Data Analysis

Database searches were conducted with the following parameters: Trypsin/P was specified as the enzyme specificity, allowing up to two missed cleavages. Carbamidomethylation of cysteine residues was set as a fixed modification, while oxidation of methionine, acetylation of protein N-termini, and deamidation of asparagine and glutamine were included as variable modifications. The minimum peptide length was set to seven amino acid residues. Mass tolerances were set at 10 ppm for precursor ions and 0.02 Da for fragment ions. For functional protein analysis and differential expression protein (DEP) identification, median normalization was performed on the raw data to minimize experimental variability. Subsequently, samples with more than 50% missing values were excluded from further analysis. Missing values in the remaining dataset were imputed using the Perseus algorithm. Differentially expressed proteins (DEPs) were defined based on the following criteria: a minimum average fold change of 2.0 (up or down-regulated) and a statistical significance level of $p < 0.05$.

4. qRT-PCR

The sequences of the gene-specific primers forqRT-PCR are listed in SupplementaryTable S2.

Table S2 The sequences of gene-specificPrimers forqRT-PCR.

Primer	Sequence (5'-3')
actin F	CCGGTCGTGACCTCACTGATTTCT
actinR	CATCAGGCAACTCGTAGCTCTTCTCG
ER lumen protein-retaining receptor F	AAACGGTCCATGTTGCTGGG
ER lumen protein-retaining receptor R	CTAACGCGGTGTGTATGTTGG

beta-amylase-1 F	GCACCTGAGAAGCTGGTGA
beta-amylase-1 R	GTGAAGGCACACATCTCTCTATC

Results

Table S3 Summary of the proteins identified in this study.

<table>
<tr><th>Sample</th><th>No. Of Identified protein</th><th>No. of Quantified protein</th></tr>
<tr><td>ZhH302E2-R1</td><td rowspan="24">5493</td><td>5056</td></tr>
<tr><td>ZhH302E2-R2</td><td>5101</td></tr>
<tr><td>ZhH302E2-R3</td><td>5084</td></tr>
<tr><td>ZhH302E3-R1</td><td>5254</td></tr>
<tr><td>ZhH302E3-R2</td><td>5227</td></tr>
<tr><td>ZhH302E3-R3</td><td>5224</td></tr>
<tr><td>ZhH302E4-R1</td><td>5131</td></tr>
<tr><td>ZhH302E4-R2</td><td>5160</td></tr>
<tr><td>ZhH302E4-R3</td><td>4967</td></tr>
<tr><td>AE15E2-R1</td><td>5120</td></tr>
<tr><td>AE15E2-R2</td><td>5101</td></tr>
<tr><td>AE15E2-R3</td><td>4930</td></tr>
<tr><td>AE15E3-R1</td><td>5149</td></tr>
<tr><td>AE15E3-R2</td><td>5128</td></tr>
<tr><td>AE15E3-R3</td><td>5133</td></tr>
<tr><td>AE15E4-R1</td><td>5003</td></tr>
<tr><td>AE15E4-R2</td><td>5023</td></tr>
<tr><td>AE15E4-R3</td><td>5036</td></tr>
<tr><td>ZhH10-R1</td><td>4512</td></tr>
<tr><td>ZhH10-R2</td><td>4598</td></tr>
<tr><td>ZhH10-R3</td><td>4541</td></tr>
<tr><td>ZhH42-R1</td><td>4771</td></tr>
<tr><td>ZhH42-R2</td><td>4781</td></tr>
<tr><td>ZhH42-R3</td><td>4780</td></tr>
</table>

Tables S4–S15are provided as Excel files.

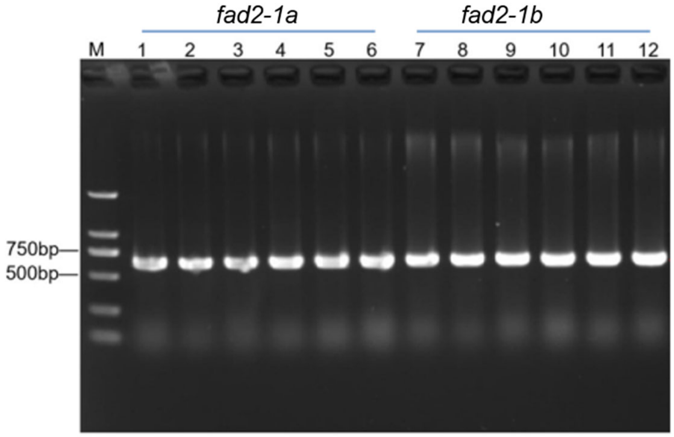

Figure S1. PCR amplification of *fad2-1* fragments (1-620 bp of *fad2-1a*, and 23-647 bp of *fad2-1b*). M, DNA marker; 1, ZhH302E2; 2, ZhH302E3; 3, ZhH302E4; 4, AE12E2; 5, AE12E3; 6, AE12E4; 7, ZhH302E2; 8, ZhH302E3; 9, ZhH302E4; 10, AE12E2; 11, AE12E3; 12, AE12E4

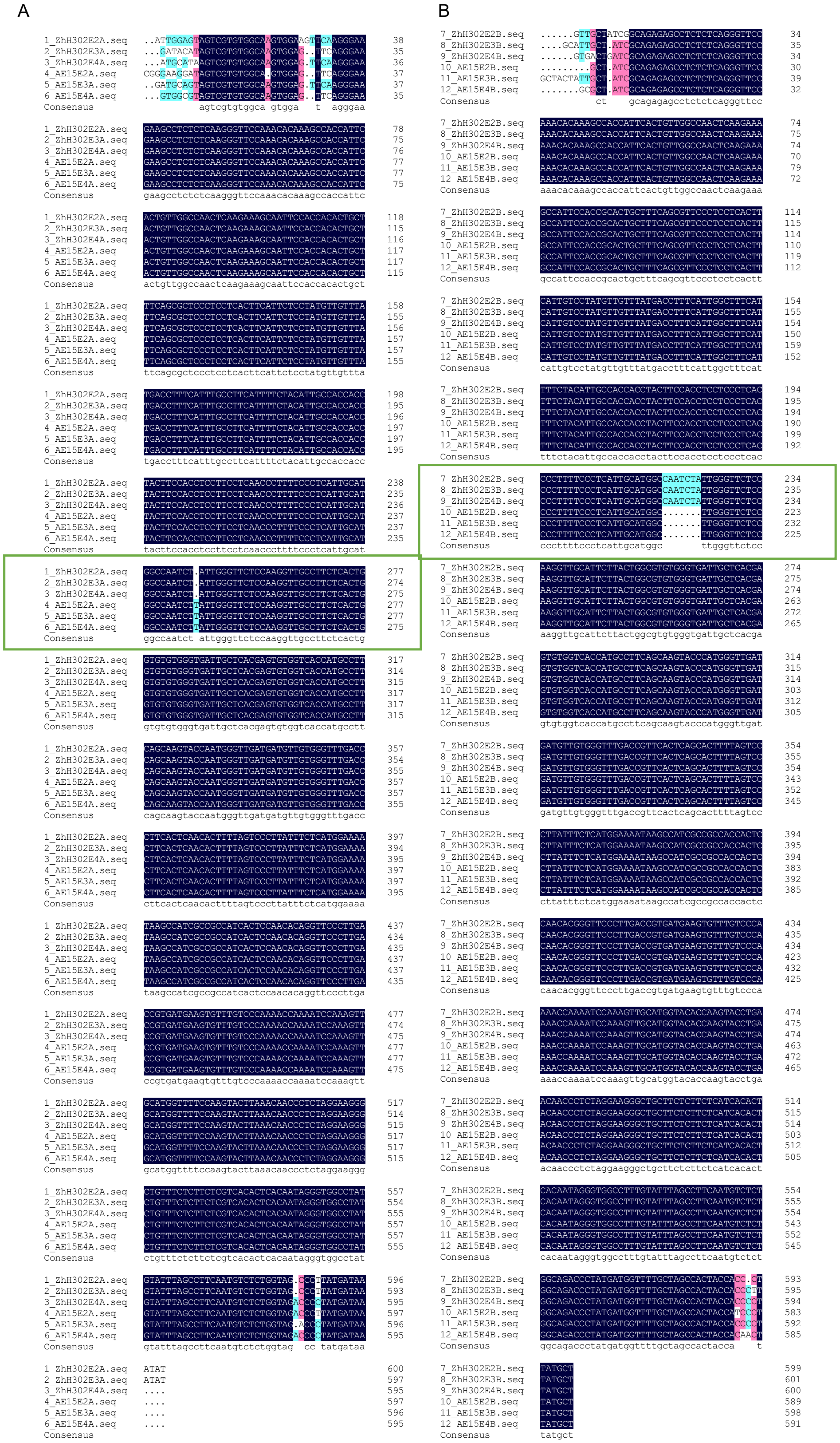

Figure S2. Sequence alignment of the *fad2-1* fragments in three consecutive generations of the *fad2-1* gene-edited soybean line AE15compared to ZhH302. A, *fad2-1a*; B, *fad2-1b*. Editing sites

of *fad2-1*were marked with green boxes.

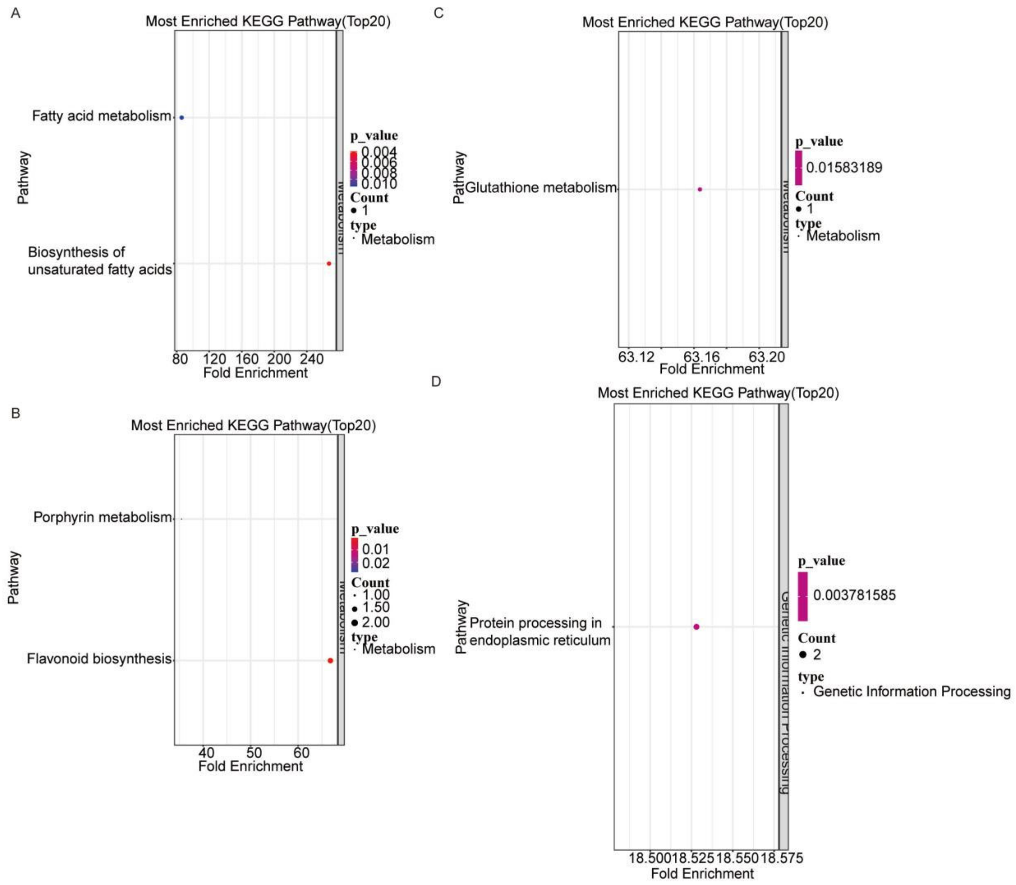

Figure S3. KEGG pathway enrichment analysis of co-differentially expressed proteins (co-DEPs) identified from pairwise comparisons among the three AE15/ZhH302 groups (A), wild-type soybean lines ZhH302E3, ZhH10, and ZhH42 (B), three consecutive generations of ZhH302 (C), and AE15 (D).
